# Dutch public health professionals’ perspectives and needs regarding citizen involvement in COVID-19 contact tracing through digital support tools: an exploratory qualitative study

**DOI:** 10.1186/s12913-022-08764-y

**Published:** 2022-11-19

**Authors:** Y. B. Helms, M. L. Stein, N. Hamdiui, A. van der Meer, R. Baron, R. Eilers, R. Crutzen, M. E. E. Kretzschmar, A. Timen

**Affiliations:** 1 National Coordination Centre for Communicable Disease Control (LCI), Centre for Infectious Disease Control (CIb), National Institute for Public Health and the Environment (RIVM), Bilthoven, the Netherlands; 2 Department of Primary and Community Care, Radboud Institute for Health Sciences, Radboud University Medical Center, Nijmegen, the Netherlands; 3 Department of Health Promotion, Care and Public Health Research Institute (CAPHRI), Maastricht University, Maastricht, the Netherlands; 4 Julius Center for Health Sciences and Primary Care, University Medical Center Utrecht (UMCU), Utrecht University, Utrecht, The Netherlands; 5 Centre for Infectious Disease Control, National Institute of Public Health and the Environment (RIVM), Bilthoven, The Netherlands

**Keywords:** Contact tracing, COVID-19, Healthcare delivery, Qualitative research, eHealth, Healthcare professionals, Patient participation

## Abstract

**Background:**

Contact tracing (CT) is an important, but resource-intensive tool to control outbreaks of communicable diseases. Under pandemic circumstances, public health services may not have sufficient resources at their disposal to effectively facilitate CT. This may be addressed by giving cases and their contact persons more autonomy and responsibility in the execution of CT by public health professionals, through digital contact tracing support tools (DCTS-tools). However, the application of this approach has not yet been systematically investigated from the perspective of public health practice. Therefore, we investigated public health professionals’ perspectives and needs regarding involving cases and contact persons in CT for COVID-19 through DCTS-tools.

**Methods:**

Between October 2020 and February 2021, we conducted online semi-structured interviews (*N* = 17) with Dutch public health professionals to explore their perspectives and needs regarding the involvement of cases and contact persons in CT for COVID-19 through DCTS-tools, in the contact identification, notification, and monitoring stages of the CT-process. Interviews were audio recorded and transcribed verbatim. A thematic analysis was performed.

**Results:**

Four main themes related to Dutch public health professionals’ perspectives and needs regarding involving cases and contact persons in CT for COVID-19 through DCTS-tools emerged from the data: ‘Distinct characteristics of CT with DCTS-tools’; ‘Anticipated benefits and challenges of CT for COVID-19 with DCTS- tools’; ‘Circumstances in CT for COVID-19 that permit or constrain the application of DCTS-tools’; and ‘Public health professionals’ needs regarding the development and application of DCTS-tools for CT’. Public health professionals seem to have a positive attitude towards involving cases and contact persons through DCTS-tools. Public health professionals’ (positive) attitudes seem conditional on the circumstances under which CT is performed, and the fulfilment of their needs in the development and application of DCTS-tools.

**Conclusions:**

Dutch public health professionals seem positive towards involving cases and contact persons in CT for COVID-19 through DCTS-tools. Through adequate implementation of DCTS-tools in the CT-process, anticipated challenges can be overcome. Future research should investigate the perspectives and needs of cases and contact persons regarding DCTS-tools, and the application of DCTS-tools in practice.

**Supplementary Information:**

The online version contains supplementary material available at 10.1186/s12913-022-08764-y.

## Background

Contact tracing (CT) is an important tool for controlling the spread of communicable diseases that transmit via (in)direct physical contact between individuals, including the ongoing SARS-CoV-2 (COVID-19) pandemic. The primary goals of CT are to identify contacts of cases (i.e., individuals with a confirmed infection) to interrupt transmission chains, and thereby protect individual and public health. In addition, the epidemiological data collected through CT are of crucial importance for surveillance and knowledge generation about communicable diseases, and for the detection of clusters of infections for which additional containment measures might be needed (i.e., outbreaks linked to specific settings, such as workplaces or healthcare facilities).

CT for COVID-19 usually takes place after an individual tests positive for COVID-19 and public health services (PHS), who are typically responsible for CT, are subsequently notified. Though the precise execution of CT for COVID-19 differs between countries, or even regions, and ‘pandemic waves’, it traditionally consists of several stages. First, in the contact identification stage, a public health professional (PHP) interviews a case to identify individuals (referred to as ‘contact persons’) who were in close physical proximity to the case during their infectious period (usually from two days before - until at most 14 days after symptom onset) and the settings in which these interactions took place. Second, in the contact notification stage, PHPs inform the identified contact persons and/or settings (e.g., through the owner of a restaurant) of their possible exposure and what CT-measures may be required to prevent onward transmission of the virus, such as testing and quarantine. Third, in the contact monitoring stage, PHPs monitor the health of contact persons (e.g., through phone calls at regular intervals) and provide them with advice and support, if needed. If contact persons test positive for COVID-19 during the monitoring period, they are required to isolate for the remaining duration of their infectious period and become new cases, with whom the CT-process is repeated [1].

Previous studies indicate that CT, in combination with CT-measures (i.e., quarantine/isolation and testing), can contribute significantly to a reduction of COVID-19 transmission, if they are executed in a timely and complete manner [2–6]. Modelling studies have estimated that CT can help to reduce the effective reproduction number of COVID-19 and thereby significantly slow down the transmission of the virus, if 60–100% of infected contact persons are identified, tested, and isolated through CT, within 0 to 4 days of symptom onset of the case to whom they were in close physical proximity (where earlier quarantine of contact persons leads to increased prevention of transmission, and vice versa) [3, 4]. However, the ‘traditional’ CT-process, as described above, relies heavily on manual labor from PHPs, who sometimes need to reach complex and large contact networks. Under pandemic circumstances, with a high and increasing daily caseload, and during ‘pandemic peaks’ in particular, PHS may not have sufficient (human) resources at their disposal to effectively facilitate CT [7].

Therefore, PHS sometimes have to scale down the execution of CT to keep up with large numbers of cases. This often entails that PHPs increasingly focus on relatively high-risk (in terms of exposure-risk and/or risk for individual or public health) individuals and settings and/or completely stop the execution of parts of the CT-process [8]. In the Netherlands, for example, PHS would scale down CT with increasing caseload (and vice versa with decreasing caseload) in seven subsequent phases. In phase 1, CT was fully facilitated by PHPs, for all cases and contact persons. In phase 1B, PHPs no longer monitored the health of contact persons and stopped following up with cases. In phase 2, PHPs still notified high-risk contact persons and settings, but left the notification of relatively low-risk contact persons up to the cases themselves. In phase 3, PHPs helped cases to identify their contact persons, but left the notification of contact persons to be conducted by the cases themselves. In phase 4, PHPs continued to collect (epidemiological) data from cases, for example regarding potential clusters of infections, but left the identification and notification of contact persons completely up to the cases. In phases 5 and 5B, PHPs further scaled down CT by collecting less and less information from cases [9].

**Fig. 1:**
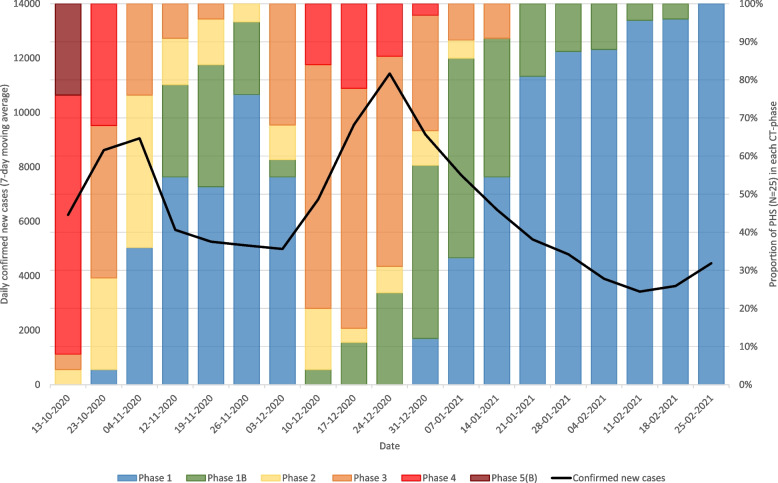
Daily confirmed new COVID-19 cases and proportion of Dutch public health services in each CT-phase. Based on contextual data regarding the weekly CT-phases of Dutch PHS that were collected and provided by each respective PHS and were shared with the Dutch National Institute for Public Health and the Environment (RIVM) by the umbrella organization of PHS in the Netherlands (GGD GHOR NL) and publicly available data regarding the daily number of confirmed COVID-19 cases in the Netherlands [10]

To illustrate, Fig. 1 shows a timeline of the daily number of confirmed COVID-19 cases (7-day moving average) and the proportion of PHS (25 in total) in each CT-phase in the Netherlands around the time that this study was conducted (between October 2020 and February 2021). We emphasize that Fig. 1 is not an indication of the effectiveness of CT in the Netherlands but illustrates how PHS responded to changing infection pressure. For example, we did not include the implementation and/or lifting of other measures to reduce transmission of COVID-19 in the Netherlands, such as lockdown and curfew.

Though the strategy outlined above makes the execution of CT more manageable from a resource-perspective, it inevitably also makes CT less thorough and comprehensive. In the Netherlands, the scaling down of CT is therefore combined with explicitly transferring responsibility in the execution of CT from PHS to cases and contact persons. This means that PHPs, depending on the degree to which CT is scaled down, request cases to identify and notify their contact persons - and contact persons to monitor their health and implement necessary CT-measures themselves [9, 11]. However, though already applied in practice, the application of this approach has not yet been systematically investigated from the perspective of public health practice. Therefore, the aim of this research was to investigate the perspectives and needs of PHPs regarding actively and more autonomously involving cases and contact persons in the traditional execution of CT for COVID-19.

In addition, we investigated if and how this approach could be facilitated by using digital tools. Since the onset of the COVID-19 pandemic, a significant amount of research has been conducted on digital tools to enhance CT. However, most research has focused on ‘proximity-based’ mobile applications that automatically and anonymously record interactions between individuals (e.g., through Bluetooth and/or GPS) and notify contact persons of cases. Though this approach has the benefit of not being critically dependent on the ability of individuals to accurately recall their contact persons, its effectiveness depends strongly on the population uptake of proximity-based mobile applications and the technology’s accuracy at recording interactions between individuals in various settings (e.g., indoors/outdoors, at home or at a restaurant) [4, 12]. In addition, many proximity-based mobile applications (including the Dutch ‘CoronaMelder’) feature a decentralized design for data storage and processing and/or minimize the collection of (personal and epidemiologically relevant) data due to privacy-related reasons. This means that such applications do not provide PHS with (useful) information for surveillance purposes and for carrying out CT to individuals and settings that are ‘missed’ by proximity-based mobile applications. As such, though proximity-based mobile applications can contribute to reducing transmission of COVID-19, they cannot replace or unburden the traditional execution of CT by PHS [12–15].

**Fig. 2:**
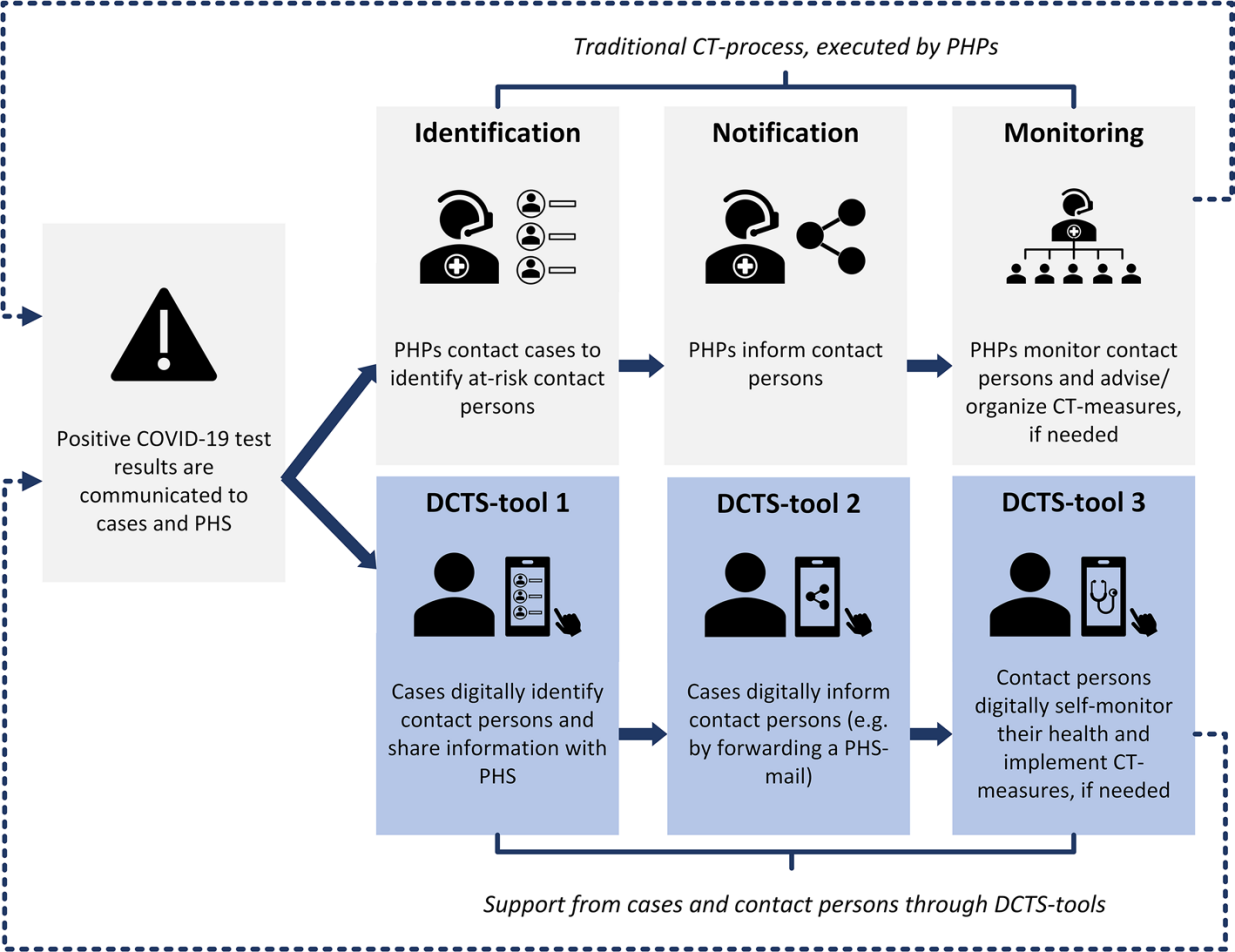
Schematic overview of digital contact tracing support tools to support the traditional CT-process

Therefore, in this research we specifically focused on digital tools that may be used by cases and contact persons to support each stage of the traditional CT-process facilitated by PHS (hence forth referred to as digital contact tracing support tools, or DCTS-tools). With DCTS-tool 1, cases can digitally collect and share their personal (health) data and their contact persons’ data with PHS in the contact identification stage; with DCTS-tool 2, cases can digitally notify their contact persons in the contact notification stage; with DCTS-tool 3, contact persons can self-monitor their health in the contact monitoring stage. See Fig. 2 for a schematic overview of the types of DCTS-tools on which we focused on in this study. Note that although we present and investigate DCTS-tools 1, 2, and 3 separately in this study, they could potentially also be combined into one or two (mobile and/or web-based) applications with multiple functionalities.

The research questions that guided this study were: 1. ‘What are PHPs’ perspectives towards actively and more autonomously involving cases and contact persons in CT for COVID-19 through DCTS-tools?’, and 2. ‘What are PHPs’ needs regarding the application and development of DCTS-tools for CT of COVID-19?’

We conducted an exploratory qualitative study among PHPs who were involved in the execution, organization, and/or coordination of CT for COVID-19 in the Netherlands.

## Materials and methods

### Study design

A cross-sectional exploratory semi-structured interview study was performed. For the most part, we followed the COREQ-checklist for interviews and focus groups to enhance this study’s methodological quality and transparency [16]. Note that some items from this checklist (e.g., member checks, repeat interviews) were not implemented due to resource and/or time constraints with the research team, as well as with the research participants.

### Study population

CT in the Netherlands is traditionally executed by PHPs (qualified public health nurses and -doctors). During the COVID-19 pandemic, many temporary CT-employees (e.g., medical students) were hired for the execution of CT to overcome personnel shortages, shifting PHPs more towards the coordination and organization of CT. However, in contrast to PHPs, temporary CT-employees are usually not specialized in public health, have very limited (or no) experience with CT in general, and have limited oversight over the CT-process in its entirety. Therefore, in this study, we primarily focused on PHPs.

### Development of the research materials

To investigate PHPs’ perspectives and needs regarding more actively and autonomously involving cases and contact persons in CT for COVID-19 through DCTS-tools, we developed a semi-structured interview guide based on the reasoned action approach (RAA). The RAA assumes that an individual’s intention to perform a certain behavior (in this study a PHP’s intention to involve cases and contact persons more actively and autonomously in CT through DCTS-tools) is determined by a set of behavioral, normative, and control beliefs, that lead into three proximal determinants: attitude, perceived norm, and perceived behavioral control [17]. In this study, we developed interview questions based on the RAA’s proximal determinants to elicit PHPs’ beliefs that underpin their perspectives and needs regarding more actively and autonomously involving cases and contact persons in CT of COVID-19 through DCTS-tools. The interview guide (translated to English) can be found in Additional file 1.

Interviews were structured into four main sections. Section one contained general questions regarding PHPs’ experiences with CT for COVID-19, how they felt about the role and participation of cases and contact persons in CT, and their perspectives on digitalization of CT. Section two contained a step-by-step presentation of Fig. 2, to introduce interviewees to the DCTS-tools of interest to this study (delivered through PowerPoint V.2102; figures were created using Microsoft Visio Professional V.2102). The DCTS-tools are based on previous research that we conducted on the application of respondent-driven detection, a snowball-like sampling method for case finding, in the context of pathogens that transmit via close (physical) contact between individuals [18, 19]. DCTS-tools were presented to interviewees in a generic fashion, to allow interviewees to deliberate freely. Section three contained questions based on the RAA’s sub-constructs, which were separately asked for all three DCTS-tools. Section four contained questions regarding interviewees’ final suggestions and thoughts regarding DCTS-tools in general.

The interview guide and the introduction of the DCTS-tools were extensively pilot tested among PHPs from the RIVM. Based on the pilot interviews, only small changes to the order of the questions were made.

### Sampling and data collection

Between October 2020 and February 2021, semi-structured in-depth one-on-one interviews were conducted with PHPs involved in the execution of CT for COVID-19, from various PHS in the Netherlands.

There are 25 PHS in the Netherlands, all of which serve different geographic regions. At the time that this study was conducted, the execution of CT for COVID-19 differed between - as well as within - PHS over time, mainly due to (regional) fluctuations in caseload (as illustrated in Fig. 1). We anticipated that the degree to which PHPs’ respective PHS would execute ‘full’, or ‘scaled down’ CT, and other PHS-specific factors related to the execution of CT, could influence PHPs’ perspectives and needs regarding actively and autonomously involving cases and contact persons through DCTS-tools. To account for this, we aimed to include at least one PHP from a PHS from each of the twelve provinces in the Netherlands, over a period of several months. In addition, we aimed to include PHPs with diverse characteristics, including age, gender, years of experience with CT (also prior to the COVID-19 pandemic), and professional background (nurse or doctor).

PHPs were identified and invited through the professional network of the RIVM, using snowball sampling and referrals (i.e., a combination of convenience and purposive sampling was used). For example, we asked regional PHS-coordinators to distribute an invitation to different PHS (for further internal distribution to PHPs) and/or to directly refer us to potential research participants. PHPs who received an invitation through their respective PHS and contacted the executive researcher (YBH) to express interest in participating in the study, and PHPs whom we were directly referred to, first received an in-depth information letter about the study via e-mail. PHPs who then wanted to participate in the study were asked to agree to the terms of the study through an online informed consent form. Finally, after having agreed to the informed consent form, PHPs were automatically redirected to a short online questionnaire through which we asked for their age, gender, professional background, province of employment, years of experience with CT, and months of experience with CT for COVID-19. The online informed consent form and questionnaire were distributed using software for respondent-driven sampling that was jointly developed by the RIVM, UMC Utrecht (the Netherlands), and the Karolinska Institute (Sweden).

Due to the social distancing measures implemented at that time, interviews were conducted online, using the web-conference software Cisco WebEx Meetings (V.40.2.14.19). Interviews were conducted by one male interviewer (YBH) and lasted approximately one hour. Interviewees received a small token of appreciation (worth approximately €10) for their participation.

### Data analysis

Interviews were audio recorded, using the recording tool available in Cisco WebEx Meetings, and transcribed verbatim. A thematic analysis (making use of open, axial, and selective coding) was conducted in MAXQDA Plus 2022 (Release 22.0.0) [20]. Coding was inductive and was guided by the research questions presented earlier. We started by analyzing if and why (research question 1), and how (research question 2) PHPs would want to involve cases and contact persons through DCTS-tools 1, 2, and 3, separately. From this, we identified overarching themes related to PHPs’ perspectives and needs regarding the application of DCTS-tools in general. To reduce subjective interpretation of the data, random selections of 13 and 5 transcripts were also coded by a second (AM) and by a third (RB) researcher, respectively. Coding was discussed until consensus was reached.

### Ethical considerations

This study was reviewed by the Medical Ethical Committee of the University Medical Centre Utrecht, who exempted this study from the need for a full medical ethical review (reference number: 20–662/C).

## Results

### Sample characteristics, data saturation and response rate

Seventeen PHPs were interviewed, representing PHS from all 12 provinces in the Netherlands. Interviewees had a median age of 39 years (IQR: 32–50) and a median of 4 years (IQR: 2.5–9) experience with CT in general. The median CT-experience for COVID-19 was 8 months (IQR: 5–9). Most interviewees were female (76.5%) and worked as a PHS-nurse (76.5%). At the time that the interviews were conducted, most interviewees’ PHS facilitated full CT (52.9%). See Table 1 for an overview of the sample characteristics.

**Table 1:** Characteristics of interviewees

Characteristics	Interviewees (*N* = 17)
Age, in years (Mdn; IQR^a^)	39 (32; 50)
Sex (%)	
• Male	4 (23.5)
• Female	13 (76.5)
Experience with CT, in years (Mdn; IQR)	4 (2.5; 9)
Experience with CT for COVID-19, in months (Mdn; IQR)	8 (5; 9)
Professional role (%)	
• PHS-Nurse	13 (76.5)
• PHS-Doctor	4 (23.5)
PHS CT-phase^b^ (%)	
• Phase 1	9 (52.9)
• Phase 1B	2 (11.8)
• Phase 2	1 (5.9)
• Phase 3	4 (23.5)
• Phase 4	1 (5.9)
• Phase 5(B)	0 (0)

^a^*Mdn* Median, *IQR* Inter-Quartile Range

^b^Phase 1: PHPs fully facilitated CT; Phase 1B: PHPs stopped monitoring contact persons and following up with cases; Phase 2: PHPs only facilitated CT for high-risk individuals and settings; Phase 3: PHPs aided cases with the identification of contact persons, but no longer notified them; Phase 4: PHPs left the identification and notification of contact persons completely up to cases; Phases 5 and 5B: PHPs collected less epidemiological data from cases

Although we did not perform an in-depth analysis in this regard, we did not encounter (signs of) relationships between sample characteristics and interviewees’ perspectives and needs towards DCTS-tools, except for ‘PHS CT-phase’, which is discussed in more detail under ‘Circumstances in CT for COVID-19 that permit or constrain the application of DCTS-tools’ in the results section.

No new (sub-)themes were identified (i.e., data saturation was reached) after the analysis of 11 interviews.

Since we relied on referrals and snowball sampling for the recruitment of PHPs, it is impossible to calculate the response rate of our study. However, all individuals to whom we eventually sent the online informed consent form and questionnaire participated in this study.

### Main themes that emerged from the data

Four main themes related to PHPs’ perspectives and needs regarding the application of DCTS-tools for CT of COVID-19 emerged from the data: ‘Distinct characteristics of CT with DCTS-tools’, ‘Anticipated benefits and challenges of DCTS-tools for CT of COVID-19’, ‘Circumstances in CT for COVID-19 that permit or constrain the application of DCTS-tools’, and ‘PHPs’ needs regarding the application and development of DCTS-tools’. Main- and sub-themes are discussed in more detail in the following sections.

#### Distinct characteristics of CT with DCTS-tools

We found that PHPs’ perspectives and needs regarding the application of DCTS-tools were generally underpinned by how they anticipated several distinct characteristics of CT with DCTS-tools, that set it apart from the traditional execution of CT by PHPs. We identified two sub-themes in this regard: ‘Uncertainty about giving cases and contact persons more autonomy and responsibilities in CT’, and ‘Digitalization can enhance CT, but may not address the complexity and interpersonal aspects of CT’. See Table 2 for illustrative quotes.

**Table 2:** Illustrative quotes related to distinct characteristics of CT with DCTS-tools

Sub-theme	Illustrative quotes
Uncertainty about giving cases and contact persons more autonomy and responsibilities in CT	“I think that all of the Netherlands is aware of CT and what it entails. I really do not think that PHS need to fully facilitate CT, and that for at least 90 % of all cases you can put them in control.” PHS-nurse, female, mid-20’s.
	“You lose a lot of control in the sense of, will cases do all those things? Will the right people get the right information? That’s a pretty big responsibility to give away. And of course, as PHS we also have a legal responsibility to identify and notify the right contact persons.” PHS-nurse, female, early 40’s.
Digitalization can enhance CT, but may not address the complexity and interpersonal aspects of CT	“We, as a PHS, are really looking into how we can make things easier and faster, the whole process of calling cases and contact persons and getting all this data into our systems in a uniform manner. And it just comes down to digitalizing these processes.” PHS-nurse, female, mid-20’s
	“Of course, this is also a chance to prepare ourselves for the future. Now we have COVID, but there will be other things for which we will have to prepare better. The systems we have now, are not good enough.” PHS-doctor, male, late-50’s
	“These [DCTS-tools] are all tools that can help. And it can work very well, as long as you apply it correctly. So, I am not against it, but I believe that we need to remember that there still is a patient behind all this technology.” PHS-nurse, female, early-50’s

##### Uncertainty about giving cases and contact persons more autonomy and responsibilities in CT

Compared to traditional CT, interviewees noted that cases and contact persons would have more autonomy and responsibilities with DCTS-tools. Interviewees had diverging perspectives on this characteristic of CT with DCTS-tools. Interviewees often felt that people in the Netherlands are relatively (compared to other communicable diseases) aware and knowledgeable of what CT for COVID-19 entails and why it is necessary, and increasingly want to be independent from PHS (e.g., because they experience the traditional execution of CT as unnecessarily controlling or patronizing). As such, they reasoned that PHPs should not always want - and have to (fully) facilitate CT. However, interviewees also frequently questioned the willingness and ability of cases and contact persons to participate in CT more autonomously, and worried that PHPs may lose control and oversight over the CT-process if cases and contact persons do not adequately do so. This was considered more problematic by interviewees who more strongly felt that it is the (legal) responsibility of PHPs to make sure that the right contact persons are identified, notified, and monitored, so to oversee and safeguard the health of the population.

##### Digitalization can enhance CT, but may not adequately address the complexity and interpersonal aspects of CT

Interviewees noted that the development and implementation of DCTS-tools require digitalization of the traditional CT-process. This was supported by most interviewees, as it was generally believed that digitalization would make CT more efficient and/or that the traditional execution of CT and the current digital infrastructure for CT were outdated and not ‘future-proof’. However, some interviewees questioned to what degree CT could – and should – be digitalized, as they worried that this could lead to neglecting its complexity and interpersonal aspects (e.g., emotional support for cases and contact persons). In addition, there were concerns that digitalization of CT may not be equally beneficial or could even be detrimental to some PHPs, cases, and contact persons, for example to those with limited digital health skills and literacy.

#### Anticipated benefits and challenges of CT for COVID-19 with DCTS-tools

Based on our separate analyses of interviewees’ perspectives and needs regarding DCTS-tools 1, 2, and 3, we found that interviewees had a seemingly positive attitude towards the application of DCTS-tools in CT for COVID-19 in general. We identified several overarching anticipated benefits and challenges of CT with DCTS-tools that strongly influenced interviewees’ attitudes. Several sub-themes were identified in this regard.

Sub-themes related to overarching anticipated benefits were ‘CT can be executed more efficiently’, in the sense that DCTS-tools could lower the workload for PHPs, accelerate the CT-process, and more cases and contact persons could be reached; ‘Cases and contact persons have more opportunities to participate in CT in a manner that best suits them’, in the sense that it may be relatively pleasant for cases and contact persons to be less dependent on PHPs for participating in CT; and ‘Enhanced quality of CT-data collection and administration’, in the sense that DCTS-tools may enhance the completeness and correctness of the data collected through CT. Sub-themes related to overarching anticipated challenges were ‘Adequate execution of CT strongly depends on the willingness and skills of cases and contact persons’, in the sense that PHPs have less control and oversight over the CT-process; and ‘Concerns about limited support and guidance for cases and contact persons in the CT-process’, in the sense that cases and contact persons may not receive adequate emotional and/or practical support in the CT-process to motivate or guide their participation.

Though interviewees had a seemingly positive attitude towards DCTS-tools in general, their attitudes differed between the separate DCTS-tools, in line with the degree to which the overarching benefits and challenges were anticipated to manifest. Therefore, below, we separately detail interviewees’ anticipated benefits and challenges regarding the application of DCTS-tools 1, 2, and 3, in the contact identification, −notification, and -monitoring stages of the CT-process, respectively. See Table 3 for an overview of illustrative quotes regarding the anticipated benefits and challenges of DCTS-tools 1, 2, and 3, in relation to the overarching anticipated benefits and challenges described above.

**Table 3:** Illustrative quotes related to anticipated benefits and challenges of DCTS-tools 1, 2, and 3

Sub-theme	DCTS-Tool 1 (Contact identification)	DCTS-tool 2 (Contact notification)	DCTS-tool 3 (Contact monitoring)
Overarching anticipated benefits			
CT can be executed more efficiently	“I think this [DCTS-tool 1] can be pretty useful. For the simple reason that I think that you shouldn’t do work that you don’t really have to do. And people can do this themselves very well.” PHS-doctor, female, late-30’s.	“The advantage is that you want people informed about their risks as soon as possible. And if it takes too long for PHS to call people, you can be one or two days late. So that speed is pretty important. This [DCTS-tool 2] can definitely help with that.” PHS-doctor, male, late-50’s.	“Maybe people think ‘I need to really have a fever to get tested’. But when they are immediately told to get tested, even with mild symptoms, you can get them to test sooner.” PHS-nurse, female, early-30’s.
Cases and contact persons have more opportunities to participate in CT in a manner how it best suits them	“What makes this [DCTS-tool 1] especially pleasant for people is that they can do it at a moment of their choice, and not at that particular moment when they are in a supermarket with screaming kids … because, of course, that is when PHS always call.” PHS-nurse, female, early-30’s.	“Not everyone wants to share their contacts with PHS, for all sorts of reasons. But they do want to personally inform their contacts. So, I think it would be good if they are given the opportunity to do so.” PHS-doctor, female, early-30’s.	“It could give a lot of people a sense of freedom, that they can do it themselves. Instead of them being watched and ‘stalked’ by PHS.” PHS-nurse, female, early 50’s.
Enhanced quality of CT-data collection and administration	“The way it is now, is that someone gives you the information over the phone. And you quickly write it down somewhere, and then you have to enter it somewhere again. So, you make mistakes sometimes. I think this [DCTS-tool 1] is much less prone to such errors.” PHS-nurse, female, mid-20’s.	“If we could get feedback on which contacts were informed, that would be really great. That would give us a more complete picture of the information transfers, but also, for example, in the numbers of contacts.” PHS-nurse, male, early-40’s.	“If PHS can also receive these data, I see added value in this [DCTS-tool 3]. That if someone registers symptoms, that we also receive that. Because in a scaled down situation, we now have no data from contact persons at all.” PHS-nurse, female, mid-20’s
Overarching anticipated challenges			
Adequate execution of CT strongly depends on the willingness and skills of cases and contact persons	“When you do this by phone, you can immediately start putting data into the systems and start calling contacts. The disadvantage when people digitally do this themselves, is that you’re always dependent on when you get their data”. PHS-nurse, female, early 40’s.	“When cases notify their own contacts, you don’t really have insights in where the transmission is happening. It means that you have less control, less grip over what is happening with the virus.” PHS-doctor, female, late-30’s.	“You are not on top of things when something changes for the contact. So, when they think ‘it’s nothing’, or ‘it’s not COVID’, you will miss them.” PHS-nurse, male, mid-50’s.
Concerns about limited support and guidance for cases and contact persons in the CT-process	“I think that a lot of people, when they see or hear their testing result, that they are concerned. They have a lot to get of their chest. So, you have to comfort or inform them before you tell them to do this.” PHS-doctor, male, late-50’s.	“I think that cases will often not, or not completely notify their contacts. Maybe they didn’t understand the instructions, or they feel very uncomfortable. They are sick and may have infected others. It can be pretty difficult when you have to go tell that to someone.” PHS-nurse, female, early-30’s	“Often people need a pep talk to maintain their motivation to stay in quarantine, for example. What are your issues and how can we solve those? That somebody thinks along and shows you understanding. That’s something you’re missing with this [DCTS-tool 3].” PHS-nurse, female, early 50’s.

##### Anticipated benefits and challenges of DCTS-tool 1 in the contact identification stage

Overall, interviewees had a positive attitude towards involving cases in the identification of contact persons through DCTS-tool 1 and anticipated that it could significantly benefit CT. See Table 3 for illustrative quotes.

**CT can be executed more efficiently**

It was frequently anticipated by interviewees that DCTS-tool 1 would lower the workload for PHPs and allow for more contact investigations to be conducted in less time, since cases can digitally collect and share information with PHS that would normally have to be requested over the phone by a PHP. To this purpose, it was often suggested that cases should not only collect their contact persons’ data, but also their personal (health) data, such as date of onset of disease, date of testing for COVID-19, and presence of underlying medical conditions.

**Cases and contact persons have more opportunities to participate in CT in a manner that best suits them**

It was often noted that, if cases would be less dependent on direct and personal contact with a PHP over the phone, they would have more opportunities to participate in CT when and how it best suits them. Interviewees felt that this may be easier and less stressful for cases, which could potentially also allow them to better remember their contact persons and visited locations.

**Enhanced quality of CT-data collection and administration**

Interviewees felt that DCTS-tool 1 could lead to the collection of more complete and uniform CT-data, with less administrative errors, especially if the CT-data collected by cases could also be automatically transferred to the case-management software routinely used by PHPs for CT at their PHS. This was considered particularly advantageous if PHPs have limited time to collect and/or enter these data themselves.

**Adequate execution of CT strongly depends on the willingness and skills of cases and contact persons**

Interviewees believed that most cases would be willing and - if instructed well - able to autonomously identify contact persons, remember in which context and setting they saw these individuals, and digitally collect and share this information with a PHP. However, it was noted that there are many ‘grey area situations’ in the interpretation and application of contact identification and exposure-risk assessment guidelines, which could make correct application of such guidelines difficult for some cases. In addition, it was anticipated that some cases may not be as thorough as PHPs, may (intentionally or unintentionally) not report contact persons (correctly), or may not feel the urgency to act (swiftly). Interviewees worried that this may sometimes lead to missing or misclassifying contact persons, or a delay in the CT-process.

**Concerns about limited support and guidance for cases and contact persons in the CT-process**

Several interviewees also worried that without personal instructions and/or motivation from a PHP, some cases may not - or inadequately - implement and adhere to important CT-measures, such as isolation. In addition, it was noted that cases may need a PHP’s support for other reasons, for example because they are concerned about their personal - or their contact persons’ health. Interviewees worried that, with some cases, these issues could lead to suboptimal participation in CT or adherence to CT-measures.

##### Anticipated benefits and challenges of DCTS-tool 2 in the contact notification stage

Interviewees had different attitudes towards DCTS-tool 2. Though several important benefits for CT were anticipated, there were concerns about the degree to which contact persons can be adequately notified without or with limited involvement of PHPs. See Table 3 for illustrative quotes.

**CT can be executed more efficiently**

It was felt that DCTS-tool 2 could significantly reduce the workload of PHPs, accelerate the contact notification process, and allow for more contact persons to be notified. Important reasons for this were that interviewees expected that it would be relatively easy for cases (compared to PHPs) to get in touch with - and digitally notify (many) contact persons, and that cases may notify contact persons of whom they do not want to share contact details with PHS, especially if they could also do this anonymously.

**Cases and contact persons have more opportunities to participate in CT in a manner that best suits them**

In addition to cases not wanting to share contact details of their contact persons with PHS, interviewees noted that many cases already personally notify (a selection of) their contact persons, regardless of how the CT-process is organized (e.g., CT-phase). As such, it was felt that some individuals may want - or even prefer to participate in CT through DCTS-tool 2, to some degree.

**Enhanced quality of CT-data collection and administration**

Several interviewees felt that DCTS-tool 2 could provide PHPs with important additional (surveillance) data, if PHPs could receive indications from cases and/or contact persons regarding if, which, and how many contact persons (especially those who were not reached/notified by a PHP) were notified by cases.

**Adequate execution of CT strongly depends on the willingness and skills of cases and contact persons**

Interviewees often noted, however, that COVID-19 can be perceived as a relatively severe disease, especially regarding individuals at elevated risk of severe illness. In addition, the CT-measures that contact persons may need to undertake (e.g., quarantine) can be very impactful. Therefore, it was anticipated that cases may feel ashamed, guilty, or judged, when personally notifying their contact persons. Interviewees worried that this may keep cases from informing (a selection of) their contact persons. Furthermore, concerns were raised about the degree to which cases would correctly notify their contact persons. For example, it was noted that cases may need to forward different CT-guidelines to different contact persons, depending on their exposure-risk. Interviewees anticipated that cases could easily make mistakes in this regard and that contact persons could be wrongfully notified as a result. As a result, interviewees worried that PHPs could lose oversight over which contact persons are (correctly) informed, and what further actions may be necessary prevent further spread of the virus.

**Concerns about limited support and guidance for cases and contact persons in the CT-process**

Interviewees were also doubtful about the ability and willingness of contact persons to adequately interpret and implement CT-measures when digitally notified by a case, instead of personally by a PHP. Contact persons may, for example, not understand what is asked from them, not take the notification seriously, or need professional/emotional support because they are concerned about their health. Interviewees worried that this may lead to sub-optimal implementation of CT-measures by contact persons.

##### Anticipated benefits and challenges of DCTS-tool 3 in the contact monitoring stage

Most interviewees seemed slightly positive towards DCTS-tool 3 and anticipated that it could somewhat benefit CT for COVID-19. See Table 3 for illustrative quotes.

**CT can be executed more efficiently**

Interviewees often anticipated that when contact persons monitor their own health through DCTS-tool 3, this would significantly lower the workload for PHPs and allow for more contact persons to be monitored. In addition, it was felt that with DCTS-tool 3, contact persons could be prompted to think about their health more often (e.g., through push-notifications at regular intervals), and be made aware of symptoms sooner. If DCTS-tool 3 could also directly refer contact persons to make a testing appointment (e.g., when they report relevant symptoms or after a specified number of days, depending on the specific guidelines for contact monitoring), this could trigger more contact persons to test, and to test sooner. It was felt that this may accelerate the CT-process and lead to more cases being identified.

**Cases and contact persons have more opportunities to participate in CT in a manner that best suits them**

Compared to phone calls from PHPs, several interviewees anticipated that self-monitoring through DCTS-tool 3 may feel less ‘controlling’ to contact persons, and that it would give contact persons more control over the CT-process. As such, it was felt that some contact persons may prefer to participate in CT through DCTS-tool 3.

**Enhanced quality of CT-data collection and administration**

It was noted by several interviewees that DCTS-tool 3 could allow for the collection of more (uniform) epidemiological data, especially if the monitoring data that contact persons collect could also automatically be transferred to their PHS’ case-management software. This was considered particularly beneficial if monitoring is not- or only limitedly facilitated by PHPs.

**Adequate execution of CT strongly depends on the willingness and skills of cases and contact persons**

Overall, interviewees believed that compared to traditional contact monitoring through phone calls by PHPs, contact persons may be relatively willing to participate in CT through DCTS-tool 3. However, some interviewees noted that there may be limited incentives for contact persons to participate in contact monitoring in general, since they often are already aware of their risk of a COVID-19 infection and the CT-measures they should undertake (e.g., testing when symptoms occur). As such, some interviewees noted that contact monitoring in general may come across as unnecessary and controlling to contact persons and questioned if – and how many contact persons would want to participate in contact monitoring at all.

It was also often anticipated that it may be difficult for some contact persons to accurately identify and interpret their COVID-19 related symptoms. As such, interviewees worried that, without a PHP’s assessment, cases may oversee or unnecessarily report symptoms, which could lead to unnecessary testing, or, on the contrary, infections remaining undetected.

**Concerns about limited support and guidance for cases and contact persons in the CT-process**

Several interviewees also worried that, with limited support and guidance from a PHP during the monitoring phase, contact persons may struggle and/or lose motivation to continue monitoring their health, or to adhere to CT-measures, such as quarantine or testing.

#### Circumstances in CT for COVID-19 that permit or constrain the application of DCTS-tools

Interviewees strongly felt that the applicability of DCTS-tools significantly depends on the circumstances under which CT for COVID-19 is performed. Hence, interviewees’ attitudes towards the application of DCTS-tools appeared conditional on these circumstances. Identified sub-themes in this regard are: ‘The ‘success’ of DCTS-tools depends on individual characteristics of cases and contact persons’, ‘DCTS-tools are especially useful when PHS have limited capacity to facilitate ‘traditional’ CT’, and ‘DCTS-tools are less applicable in complex and/or impactful settings in CT’. See Table 4 for illustrative quotes.

**Table 4:** Illustrative quotes related to circumstances in CT that permit or constrain the application of DCTS-tools

Sub-theme	Illustrative quotes
The ‘success’ of DCTS-tools depends on individual characteristics of cases and contact persons	“The question is, what can you let people do themselves? I find this a difficult question, because it is very dependent on the specific individual.” PHS-nurse, female, early-30’s.
	“I think that PHPs should make an assessment each time: ‘with this person we will use it and with this person we won’t.” PHS-nurse, female, mid-20’s.
DCTS-tools are especially useful when PHS have limited capacity to facilitate ‘traditional’ CT	“The situation in which we were a while ago, that we just couldn’t make it to inform all the contacts… In that kind of situation this [DCTS-tool 2] can be useful.” PHS-nurse, female, late-20’s.
	“For me this [all DCTS-tools] is not only to solve the time pressure and capacity issues. I would want this anyway because I think it’s just a lot more efficient. It’s just a new way of how we deal with infections together. So, I think this is something good, also when we do have time.” PHS-nurse, female, early-50’s.
DCTS-tools are less applicable in complex and/or impactful settings in CT	“Maybe someone works at a large business, or with migrants, or at a care facility, or something like this, where you can potentially have a large outbreak. Then you need more control, and you have a lot of factors that are a little bit different than usual, that some digital application cannot consider.” PHS-doctor, female, early-30’s.

##### The ‘success’ of DCTS-tools depends on individual characteristics of cases and contact persons

Interviewees felt that the ‘success’ of DCTS-tools would significantly depend on cases’ and contact persons’ individual characteristics, such as access to digital technologies, digital skills and (health) literacy, language skills, health status, motivation and skills to participate in CT, need for professional support and guidance, exposure-risk, and risk of severe illness. Therefore, most interviewees suggested that participation in CT through DCTS-tools should be ‘optional’, or that it should be decided on a case-by-case basis whether DCTS-tools are applied or not.

##### DCTS-tools are especially useful when PHS have limited capacity to facilitate ‘traditional’ CT

The degree to which PHS have sufficient capacity available to facilitate traditional CT was frequently discussed by interviewees in relation to their attitude towards DCTS-tools. Most interviewees indicated that they may be more inclined to apply DCTS-tools when there is insufficient capacity at their PHS to (fully) facilitate traditional CT. This was particularly true for the notification of contact persons (DCTS-tool 2), which was often considered relatively burdensome for cases and contact persons, and the stage at which PHPs may lose the most oversight and control over the CT-process. Nevertheless, several interviewees were open to the application of all DCTS-tools, regardless of the available PHS-capacity, if implemented with adequate ‘checks and balances’.

##### DCTS-tools are less applicable in complex and/or impactful settings in CT

Most interviewees considered DCTS-tools to be less applicable in relatively complex and potentially impactful settings, such as outbreaks in care facilities or workplaces. It was felt that such settings often affect relatively many individuals, are more sensitive in nature, and may pose a relatively large risk to individual and public health. In addition, it was noted that in complex and impactful settings the application of CT-guidelines and measures, and the communication thereof, often require careful tailoring. Therefore, interviewees generally felt that the responsibility for the execution of CT in such settings should remain largely with PHPs.

#### PHPs’ needs regarding the application and development of DCTS-tools for CT

Interviewees expressed various needs regarding the development and application of DCTS-tools in CT for COVID-19. Mostly, interviewees’ needs were related to overcoming DCTS-tools’ anticipated challenges, whilst maintaining their anticipated benefits. As such, interviewees’ (positive) attitudes were, to a large extent, also conditional on the fulfillment of their needs in the application and development of DCTS-tools. Five sub-themes related to PHPs’ needs were derived from the data: ‘Flexible integration of DCTS-tools in the traditional CT-process’, ‘Opportunities for automatic data transfer between DCTS-tools and PHS case-management software’, ‘Usage indications and communication through DCTS-tools’, ‘Easy to use and low-effort DCTS-tools’, and ‘Adequate data security and privacy protection’. See Table 5 for illustrative quotes.

**Table 5:** Illustrative quotes related to PHPs’ needs regarding the application and development of DCTS-tools for CT

Sub-theme	Illustrative quotes
Flexible integration of DCTS-tools in the traditional CT-process	“Of course, you are going to go over it [data provided by a case in the contact identification stage] to validate or verify it. Also, because the person may have forgotten something, or may remember something during the conversation. I would find it wrong to leave it to that person completely.” PHS-nurse, male, early-30’s.
	“You could also split it up, that people inform some contacts and PHS the others. I can imagine that people don’t mind telling their own family. But colleagues at work might be a bit more difficult. You could just have the PHS tell those if people don’t want to.” PHS-doctor, male, late-50’s.
	“Sometimes we cannot quickly inform all contacts, sometimes you can’t reach people. So, you can use it as an addition, that people have the information relatively soon and know what is expected of them. But that you still contact them to assess the specific situation and check if everything is clear.” PHS-nurse, female, late-20’s.
	“Maybe this would free up some time to provide a more tailored approach. That you can continue to call people [in the contact monitoring stage] who want to get called, and let people fill out an app if they want to fill out an app.” PHS-doctor, female, late-30’s.
Opportunities for automatic data transfer between DCTS-tools and PHS case-management software	“I think nobody is looking forward to a stand-alone system that requires you to move information back and forth between records. That’s prone to errors, of course. So, if possible, an automatic connection seems ideal to me.” PHS-nurse, female, early-30’s.
	“In the current situation, we don’t even have time to identify all the contacts. But if this is automated, the quality of CT could improve. This would be very useful when there are many infections and it’s very busy, because it’s good to collect that data anyways.” PHS-doctor, female, late-30’s.
Usage indications and communication through DCTS-tools	“You lose some oversight about whether or not people are informed, do people have symptoms? Maybe, with all digital opportunities, you can let people tick a few boxes to indicate to us ‘I have read this, I know what to do, I have symptoms yes or no’. That way you still have some feedback.” PHS-nurse, female, early-30’s.
	“People like the feeling that there is a PHP behind all this who can advise them when they have questions. So, I think that is something you need to offer, that someone can contact a PHP very low-key, maybe even directly through these digital applications.” PHS-doctor, female, late-30’s.
Easy to use and low-effort DCTS-tools	“It should just be user friendly, also considering elderly people and people who are less digitally skilled, who have less language skills. So, think about the language level you use and have other languages available. It should also work on older devices, you know, those kinds of things.” PHS-doctor, male, late-50’s.
	“It should just not be a complex and very different system for us [PHPs]. I have noticed that people are reaching their digital limits, with all the systems we have to use for all sorts of different things.” PHS-nurse, female, early 50’s.
Adequate data security and privacy protection	“I think that the security and protection of people’s personal data is crucial, that needs to be guaranteed if you want to implement this [DCTS-tools].” PHS-nurse, male, early-30’s.

##### Flexible integration of DCTS-tools in the traditional CT-process

Interviewees strongly felt that DCTS-tools should be integrated in the traditional CT-process in a manner that allows PHPs to remain personally involved in the execution of CT, so to maintain oversight and control over the CT-process, and to support and guide cases and contact persons if necessary. In addition, this would allow PHPs to flexibly apply DCTS-tools with respect to the previously outlined circumstances under which DCTS-tools can be applied (e.g., characteristics of individual cases and contact persons).

In the contact identification stage, most interviewees suggested that cases could start to collect their personal – and their contact persons’ data before they are contacted by a PHP for CT, preferably immediately after they receive their positive COVID-19 test result. When a case is then contacted by a PHP (as usual), they can jointly review the collected data. This would allow PHPs to ask follow-up questions to avoid missing or misclassifying contact persons and/or potential clusters, and to check if cases require further support.

Similarly, most interviewees suggested that cases should consult a PHP before digitally notifying their contact persons. This would allow PHPs to discuss with cases who will notify whom (e.g., based on motivation, exposure-risk, or a combination thereof), motivate them if necessary, and support them in correctly notifying their contact persons. Alternatively, several interviewees suggested that cases could notify their contact persons in advance - rather than a PHP, using more general and short notification messages.

Interviewees felt less strongly that PHPs need to remain personally involved in the contact monitoring stage. It was often suggested that PHPs could leave the monitoring up to the contact persons who prefer to do so themselves, which would give PHPs more time to maintain personal contact with contact persons who do not want to – or cannot (digitally) self-monitor their health.

##### Opportunities for automatic data transfer between DCTS-tools and PHS case-management software

Interviewees often stressed the importance of a link for direct and automatic data transfer between DCTS-tools and the case-management software used at PHS. This would reduce the amount of (double) manual administration required by PHPs, reduce the likelihood of administrative errors (e.g., misspellings or missing data), and allow for continued and uniform collection and administration of CT-data when PHS have limited capacity for CT. These were considered major potential advantages by interviewees, since they could further lower the (administrative) workload for PHPs, allow PHPs to maintain more oversight over the CT-process, and enhance the quality (i.e., completeness and correctness) of CT-data.

##### Usage indications and communication through DCTS-tools

To oversee the CT-process and retain opportunities to support and guide cases and contact persons, two main suggestions were made. First, several interviewees felt that PHPs should receive indications of participation in CT from cases and/or contact persons through DCTS-tools. For example, with DCTS-tool 2, contact persons could be asked to indicate to PHS that they received a digital PHS letter and understood what is asked from them, or need further (personal) support. Second, some interviewees suggested that cases and contact persons who use DCTS-tools should have opportunities to reach out to PHS for support, for example through a priority PHS-phone number, e-mail, or chat.

##### Easy to use and low-effort DCTS-tools

Since DCTS-tools depend on actions from cases and contact persons, interviewees frequently stressed that DCTS-tools should be easily accessible and easy to use for a variety of individuals. To this purpose, it was suggested that DCTS-tools could be made available both as web- and mobile applications, in different languages, and that information and instructions (including PHS-letters) should be made available in simple written and audio-visual formats. Several interviewees noted that DCTS-tools should also be easy to use for PHPs. For example, it should be easy to transfer data to the PHS case-management software, and information, instructions, and questions for cases and contact persons should be easily adaptable.

##### Adequate data security and privacy protection

It was widely anticipated among interviewees that concerns about data security and privacy protection among cases, contact persons, and PHPs, could pose as significant barriers to the use of DCTS-tools. Therefore, interviewees stressed that these aspects should be guaranteed in the development of DCTS-tools.

## Discussion

### Main findings

In this study, we asked Dutch PHPs if, why, and how cases and contact persons could support the execution of CT for COVID-19 by PHPs through digital contact tracing support tools (DCTS-tools). Four main themes related to PHPs’ perspectives and needs were identified: ‘Distinct characteristics of CT with DCTS-tools’, ‘Anticipated benefits and challenges of CT with DCTS-tools’, ‘Circumstances in CT for COVID-19 that permit or constrain the application of DCTS-tools’, and ‘PHPs’ needs regarding the application and development of DCTS-tools’. PHPs seem to have an overall positive attitude towards the application of DCTS-tools, in all stages of the CT-process. It was anticipated that DCTS-tools can have important benefits for CT, namely: more efficient CT, more opportunities for cases and contact persons to participate in CT in a manner that best suits them, and enhanced quality of data collection and administration. However, several challenges were also anticipated, namely: adequate execution of CT strongly depends on the willingness and skills of cases and contact persons, and limited support and guidance for cases and contact persons in the CT-process. PHPs appear slightly more positive about DCTS-tool 1 in the contact identification stage, and DCTS-tool 3 in the contact monitoring stage, as interviewees were more worried about the anticipated challenges of DCTS-tool 2 in the contact notification stage. Notably, PHPs’ attitudes seem conditional on the circumstances under which CT is performed, and the fulfillment of their needs in the development and implementation of DCTS-tools.

### Relevance of findings and suggestions for further research

Our findings are largely in line with results from a previous study with a similar set-up and similar aims that we conducted among Dutch PHPs before the COVID-19 pandemic, in the context of mumps, shigella, and scabies [18]. However, in the previous study, we found that Dutch PHPs were less inclined to give cases and contact persons more autonomy and responsibility in CT in the context of communicable diseases that were perceived as relatively severe for individual and public health, as COVID-19 usually was at the time that the interviews for this study were conducted.

The current study indicates that this observed discrepancy is, at least to some degree, related to the scarcity of human resources at Dutch PHS to fully facilitate CT, especially during pandemic peaks, and the unprecedented magnitude of the COVID-19 crisis. For example, PHPs noted that DCTS-tools could provide opportunities to continue to identify, notify, and monitor contact persons, when these aspects of CT can no longer be facilitated by PHPs. In addition, interviewees noted that, compared to other communicable diseases, cases and contact persons are more aware and knowledgeable about CT for COVID-19, and about what is expected of them in that regard. Our results indicate that this makes it ‘easier’ for PHPs to rely on cases and contact persons in the execution of CT. This may potentially also be true for CT with DCTS-tools during future outbreaks of communicable diseases, and under non-pandemic circumstances, if general knowledge and awareness about CT is retained among the public to some extent beyond the COVID-19 pandemic. We emphasize, however, that this suggestion requires further in-depth exploration.

Since DCTS-tools rely on actions from cases and contact persons, PHPs’ perspectives and needs regarding DCTS-tools strongly depend on their trust in the willingness and ability of cases and contact persons to participate in CT through DCTS-tools, in a timely and complete manner. As of now, however, insights into this topic are scarce. Studies in the field of patient referral and expedited partner therapy for sexually transmitted diseases have found that notification rates and treatment outcomes can be improved by giving cases and contact persons more responsibilities and autonomy in the identification and notification of contact persons [21]. However, the identification and notification of contact persons from sexual networks is markedly different to the identification and notification of contact persons from (close) contact networks (e.g., in terms of exposure-risk assessment guidelines and stigma associated with sexual contact that cases and contact persons may experience). One study conducted during the COVID-19 pandemic found that contact persons could effectively be identified through self-led online interviews, indicating that cases may indeed have the potential to contribute to CT for COVID-19 in this regard [22]. However, this study was performed in an experimental setting (not in the context of CT) with healthy adults, which may not accurately reflect the circumstances of CT in practice. For example, previous research indicates that participation in CT generally suffers from mistrust in public institutions and privacy concerns, which may be especially challenging issues in the context of DCTS-tools that rely on relatively autonomous actions from cases and contact persons [23]. In addition, whilst digital tools may generally make CT easier and more accessible for some individuals, they may pose additional barriers for individuals who are less digitally skilled or literate [24]. Therefore, we conclude that further research is necessary to identify benefits, challenges, and needs regarding DCTS-tools from a ‘citizen’s perspective’. To this purpose, we are currently conducting a study (with a similar design and purpose to this study) among Dutch citizens. In addition, we plan to conduct small pilot studies, in which we will assess the conditions under which cases are willing and able to identify and notify their contact persons in a timely and complete manner.

### Strengths and limitations

One key strength of this study is that we conducted interviews with PHPs who were directly involved in the organization, coordination, and execution of CT during the COVID-19 pandemic, around the second pandemic peak in the Netherlands. Therefore, this study provides unique and valuable insights in the perspectives and needs of PHPs regarding the involvement of cases and contact persons in CT through DCTS-tools in a pandemic context. As such, we believe that this study is particularly relevant for public health practitioners and for the development of preparedness and response plans for (future) outbreaks of communicable diseases that transmit via (close) physical contact between individuals.

Another strength is that we interviewed a relatively diverse sample of PHPs. Therefore, we believe that we captured a broad variety of perspectives and needs from Dutch PHPs. Nevertheless, since we used a combination of purposive and convenience sampling, it should be kept in mind that we may have recruited PHPs with an above average interest or motivation to participate in the study, which may have (positively) biased our findings regarding the application of DCTS-tools. We are currently performing a quantitative follow-up study to assess PHPs’ attitudes towards DCTS-tools more thoroughly, in a larger population of PHPs.

An important limitation to our study is that our findings should primarily be interpreted in a pandemic context, where PHS-capacity for CT is limited, and where widespread circulation of a pathogen has already occurred. The limited availability of medical interventions (e.g., vaccines) to control the outbreak and the specific epidemiological characteristics of COVID-19, including its severity and infectiousness, at the time that this study was performed (the Alpha-variant becoming dominant in the Netherlands) should similarly be considered in the interpretation of our findings. Furthermore, since this study was conducted in the Netherlands, our findings may be more relevant to countries with similar characteristics, including, for example, the organization of the overall CT-system and the CT-protocols in place, and the degree of digitalization of the population (in 2018, 98% of Dutch households had access to broadband internet and 84% of the Dutch population had internet access through their mobile devices outside home or work) [25]. It may be that PHPs’ perspectives and needs regarding DCTS-tools differ in the context of a relatively new and/or isolated outbreak, when outbreak control and knowledge generation more critically rely on CT, when CT is performed for pathogens with different epidemiological characteristics (e.g., the currently dominant Omicron-variant), and/or in less digitalized countries. Nevertheless, in a similar study that we conducted before the COVID-19 pandemic, we found very similar anticipated benefits, barriers, and needs of Dutch PHPs, but in a different (non-pandemic) context and for different communicable diseases [18]. Therefore, we believe that our findings are, at least to some extent, still relevant to other contexts, and that DCTS-tools may also be of added value to CT in general (e.g., from an efficiency and/or public engagement perspective).

### Recommendations for implementation of DCTS-tools in practice

Internationally, CT for COVID-19 (and other communicable diseases) may increasingly depend on (autonomous) actions from cases and contact persons when resources for CT are scarce [8, 26]. DCTS-tools to support the execution of CT by PHPs, such as those discussed in this study, may be particularly useful under such circumstances [27]. We were, however, unable to find detailed descriptions or evaluations of DCTS-tools that allow cases and contact persons to contribute to the traditional execution of CT by PHPs in scientific literature. As such, we believe our study provides particularly useful insights for the development and implementation of DCTS-tools, as well as for readily existing DCTS-tools, that rely on actions from cases and contact persons in CT.

Based on our results, it appears crucial, from a public health practice perspective, that DCTS-tools are integrated in CT-protocols in a manner that allows PHPs to maintain some oversight and control over the CT-process. Therefore, in line with previous research, we suggest that DCTS-tools should be used in support of - rather than instead of - traditional CT, so that PHPs can remain personally involved to ‘guide’ the CT-process, or to motivate and support cases or contact persons, if necessary [12, 13, 28]. For example, in the contact identification stage, cases could be asked to start to collect their personal and their contact persons’ data using DCTS-tool 1, immediately after they receive a positive test result. When cases are then contacted by a PHP for CT (as usual), the collected data can be automatically transferred to PHS, after which a case and a PHP can jointly review the data. For cases who do not use DCTS-tool 1, the identification of contact persons can be carried out as usual. In the contact notification stage, cases and PHPs could jointly decide which contact persons are notified by whom and/or cases could quickly notify contact persons in advance of - rather than instead of – a PHP, using DCTS-tool 2. Similarly, in the contact monitoring stage, PHPs and contact persons could jointly decide if DCTS-tool 3 should be used or not. We emphasize, however, that the precise integration of DCTS-tools in CT-protocols requires further (context-specific) tailoring, and careful consideration of relevant standards for data security and privacy protection (e.g., the General Data Protection Regulation). Finally, for the purposes of this study, we treated DCTS-tools 1, 2, and 3 as separate entities. However, we would like to note that it may be beneficial for practical reasons to combine the DCTS-tools into one or two mobile and/or web-based applications (e.g., so that cases and contact persons do not have to download/use multiple applications).

### Conclusions

Dutch PHPs seem positive towards giving cases and contact persons more autonomy and responsibility in CT for COVID-19 through DCTS-tools. However, PHPs’ attitudes appear to be conditional on the circumstances under which CT is performed and the fulfillment of PHPs’ needs in the development and application of DCTS-tools. In future research, we will investigate the perspectives and needs of cases and contact persons on DCTS-tools for COVID-19, and conduct pilot studies to investigate the application of DCTS-tools in practice.

## Supplementary Information

**Additional file 1.** Interview guide.docx

## Data Availability

The data supporting the findings from this study are available from the corresponding author on reasonable request.

## Abbreviations

CT: Contact tracing
COVID-19: SARS-CoV-2
PHP: Public health professional
PHS: Public health service
DCTS-tool: Digital contact tracing support tool
