# Supplementary material for: Dutch public health professionals’ perspectives and needs regarding citizen involvement in COVID-19 contact tracing through digital support tools: an exploratory qualitative study
Source: BMC Health Serv Res. 2022 Nov 19;22:1378. doi: 10.1186/s12913-022-08764-y (PMC9675960; doi:10.1186/s12913-022-08764-y)
Supplement: Supplementary file 1 — Additional file 1. Interview guide.docx [file 12913_2022_8764_MOESM1_ESM.docx]

**Additional File 1. Interview guide (translated from Dutch to English).**

**‘Digital support from cases and contact persons in the execution of COVID-19 contact tracing’**

1. **INTRODUCTION**

My name is … and I am doing this research as a PHD-student from the National Coordination Centre for Communicable Disease Control of the National Institute for Public Health and the Environment and the University of Utrecht. As you may have read in the information letter, this research focusses on novel digital methods to involve Dutch citizens more actively in infectious disease control. More specifically, we focus on digital methods to support contact tracing for COVID-19, by more actively involving cases and their contacts in the execution of contact tracing.

Firstly, I would like to kindly thank you once more for your time and participation in this research. We are very aware of the work pressure at Dutch public health services at the moment. We really appreciate your effort.

- Could you please briefly tell me something about yourself and your work at public health services?
- Before we continue, I would like to ask you if you have any remaining questions for me?
- Was everything about the informed consent clear to you?

Before we start the interview, I would like to remind you that you can always ask me questions during the interview or stop the interview if you do not want to continue, for whatever reason. I also want to emphasize that there are no wrong answers, we are only interested in your ideas and opinions. The interview will take about one hour.

I will first ask you a couple of general questions about the execution of contact tracing in your experience, and what you feel is going well, and not so well. Then, we will discuss several ways through which cases and contact can potentially be involved in contact tracing, and what this could look like.

- Do you have any remaining questions before I start the recording?

**<Start the Recording>**

1. **GENERAL EXPERIENCES WITH COVID-19 CONTACT TRACING**

| Current situation and perception of COVID-19 contact tracing practices | - How have you generally experienced the execution of contact tracing for COVID-19 so far? - Could you tell me a bit more about some of the things that you feel have been going particularly well, or bad?   - Why do you think so? |
| --- | --- |
| Opportunities to improve COVID-19 contact tracing in general | - How do you think that the execution of contact tracing for COVID-19 may be improved in the future?   - Could you please elaborate? - Do you feel like you could personally use support in some form in the execution of contact tracing for COVID-19?   - If yes, how?   - If no, why not?? |

- 1. **GENERAL ROLE OF CASES AND CONTACT PERSONS IN COVID-19 CONTACT TRACING**

| Current situation and perception of role of cases and contacts in COVID-19 contact tracing | - How do you generally experience the participation and cooperation from cases and contacts in contact tracing for COVID-19?   - What do you feel is going well in this regard?   - What do you feel is going not so well in this regard?   - In your opinion, has this changed throughout the pandemic?     - If yes, what has, and why do you think it has? |
| --- | --- |
| General feelings about more actively involving cases and contacts in COVID-19 contact tracing | - Do you feel like cases and contacts could potentially have a more active role in the execution of contact tracing for COVID-19?   - If yes, why and how?   - If no, why not? |

1. **INTRODUCTION TO ‘DIGITAL CONTACT TRACING SUPPORT TOOLS ‘**

Before the next questions, I would like to introduce you to several options through which cases and their contacts could support the execution of contact tracing for COVID-19, using digital tools. To illustrate these options, I will share my screen and show you some images.

**<Open PowerPoint (containing a step-by-step build-up of Figure 1, as presented below) and share scree**


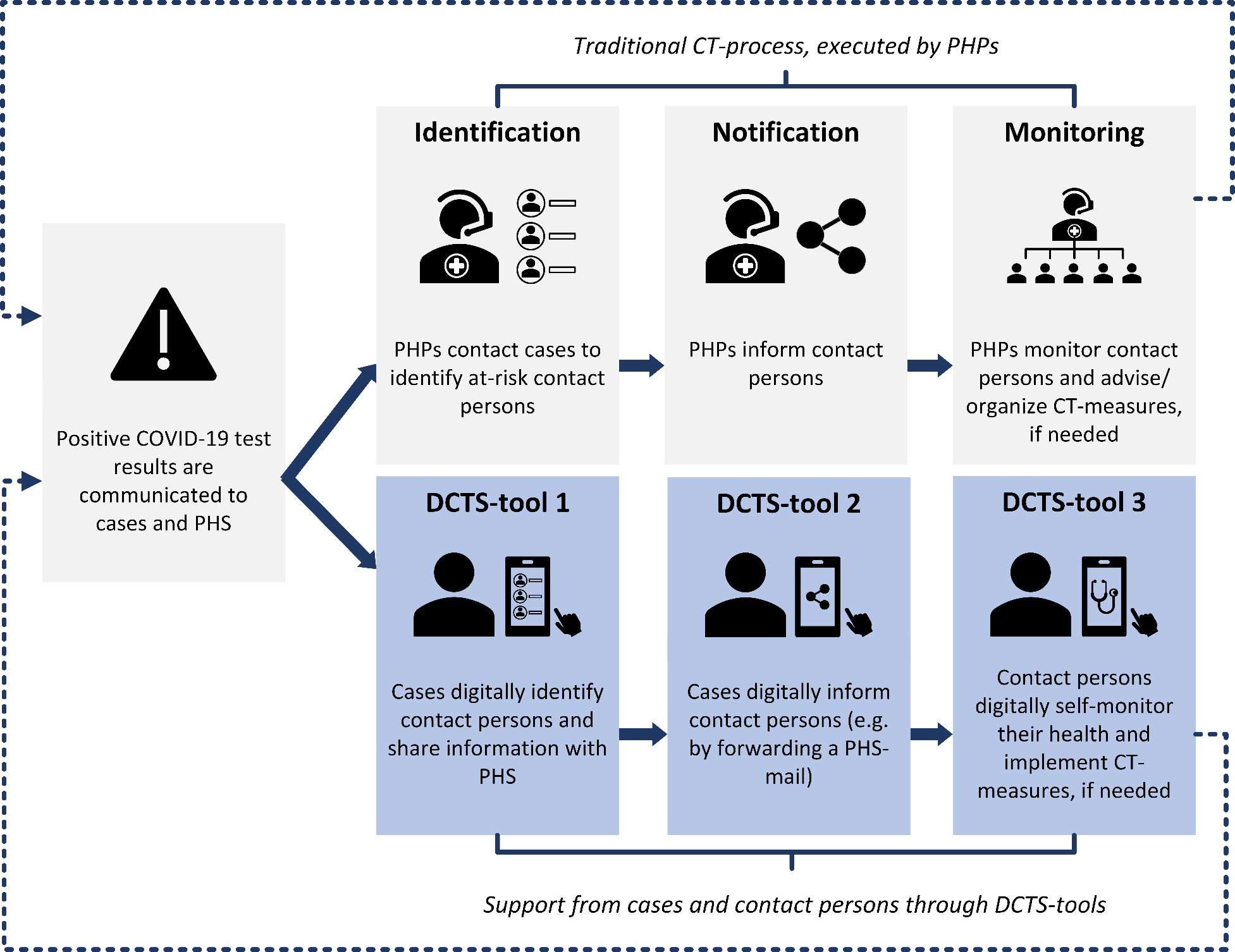
**Figure 1.** Schematic overview of DCTS-tools 1, 2, and 3 to support the traditional CT-process

Usually, the contact tracing process starts when someone tests positive for COVID-19 and the test result is communicated to public health services, and sometimes also to the case directly. Normally, a public health professional then reaches out to the case by phone, to collect the case’s personal health information, and information of his/her contacts. The public health professional then reaches out to all – or some of the contacts, depending on the situation, to inform them of their exposure, potential risks, and what measures may be necessary to prevent further spread of the virus. After this, the contact persons are monitored, for example through a phone call every x-number of days. If contacts test positive for COVID-19 during the monitoring stage, the process starts over again.

So, for the main steps in this process, there are a few potential options for cases and their contacts to support contact tracing through digital tools. For example, with **tool 1,** cases could potentially digitally collect their own personal health data and their contacts’ data, and share this information with public health services, after they receive their positive test result. With **tool 2,** cases could then digitally notify their own contact persons, for example by forwarding an official public health service letter. Finally, with **tool 3,** contacts could digitally monitor their own health and, for example, get tested if this is necessary.

The next questions are about tools 1, 2, and 3, which we will discuss one-by-one.

- Was this introduction clear to you?
- Do you feel like you understand what tools 1, 2, and 3 do, and how they are meant to support contact tracing?
- Do you have any questions before we move on to the next questions?

Then we will move on to the next questions. Again, I would like to emphasize that there are no right or wrong answers. I am only interested in your thoughts and opinions.

1. **QUESTIONS TO ELICIT PERSPECTIVES REGARDING TOOLS 1, 2, AND 3**

**<Ask all questions below for tool 1 first, then for tool 2, and then for tool 3>**

| Attitude | - What is your first impression of tool X?   - What do you think would be advantages of using tool X for contact tracing of COVID-19?     - Could you elaborate on that please?   - What do you think would be disadvantages of using tool X for contact tracing of COVID-19?     - Could you elaborate on that please? - Do any other things come to your mind when you think about using tool X for contact tracing of COVID-19? |
| --- | --- |
| Needs and application in practice | - Suppose that the public health service where you work, would start using tool X for contact tracing of COVID-19.   - What do you think this could/should look like?   - What would you need to work with tool X? |
| Perceived behavioral control | - Are there any factors/circumstances that would make it easier for you to use tool X?   - If yes, which, and why?   - If no, why not? - Are there any factors/circumstances that would make it more difficult for you to use tool X?   - If yes, which, and why?   - If no, why not? |
| Social Influence | - How do you think that others (e.g., co-workers, cases, contacts) would feel if you would use tool X in contact tracing for COVID-19?   - Do you feel like there would be certain (groups of) people who would be against this?   - Do you feel like there would be certain (groups of) people who would support this?     - How do you feel about this? |
| Intention | - Suppose that you would have the opportunity to use tool X for contact tracing of COVID-19 in real life.   - Do you think you would want to use it in practice?   - Why? |

1. **CLOSING QESTIONS**

| Prioritizing tools 1, 2, and 3 | Suppose that you would be able to choose the tools that you could (not) use for contact tracing of COVID-19.   - Which would you want to use most likely? - Which would you want to use least likely?   - Could you elaborate on this? |
| --- | --- |
| Other suggestions and closing | - Do you have any other final suggestions regarding tools 1, 2, or 3? - Do you have any other remarks or thoughts before we finish the interview? |

I have come to the end of my questions. I would like to thank you very much for your time. If there are no further points from your side, I will now end the recording.
